# Women in Factories

**Published:** 1920-12-04

**Authors:** 


THE PUBLIC WELL-BEING—(continued).

### Women in Factories.

The debate which took place in the House of Commons last week and this week on the new clause in the Women, Young Persons, and Children Employment Bill, which sanctions the employment of women and young persons in shifts, offered a timely commentary on our own references in an earlier issue to the subject of women in industry. The clause is one of doubtful phraseology, which empowers the Secretary of State to authorise such employment in any factory or workshop between the hours of six in the morning and ten in the evening on any week-day, except Saturday, and between the hours of six and two on Saturday, in shifts averaging for each shift not more than eight hours a day.

It is quite evident that this clause from two points of view thoroughly alarmed members in all parts of the House. In the first place it seemed to be conferring a dangerous and undesirable responsibility upon a single Minister, and in the second place it appeared to be going contrary to the whole trend of modern legislative thought in regard to the employment of women and young people. Major Ernest Gray, a Conservative member, delivered a particularly impassioned speech in which he championed mothers and girls who under our industrial system are condemned to work in factories. "Have any of you," he asked, "listened to the clatter of the clogs early in the morning in a Lancashire town when you yourselves were lying comfortably in bed? Has a man, after his eight hours in the factory, to prepare meals for the household, or go to the wash-tub? And what of the home life of the women with grown-up children under the two-shift system; some members of the house leaving at 5.30 A.M. and others at 1.30 P.M.?" He protested against the necessities of wartime being used in order to make an inroad into the administration of the Factory Acts.

On the whole, however, the intention of the clause appears to have been misapprehended, and Mr. Inskip, on behalf of the Government, showed that there is no intention of applying the two-shift system to the textile trade; that there will be no interference with the working of the new Education Act; that the effect of the clause will be to reduce the hours of women workers to forty-one and, in some cases, thirty-eight per week—a very striking advance, if it comes true—that the 15,000 women affected are almost all within those categories of special industries as to which the Labour party's two spokesmen, Dr. Marion Phillips and Miss Symonds, agree that the system of two shifts can be justified; and that the rejection of the clause would mean the dismissal of thousands of women, and in consequence the dismissal of a large number of skilled male workers.

These points were strongly made. At the same time, if the clause is to be made satisfactory to those who are trying to secure conditions for women workers in the factory that can be made consonant with expert medical and sociological opinion, pressure should be brought to bear on the Government to remove the apparently unlimited power given to the Home Office to sanction the system wherever it pleases; and it is equally not to be denied that women and young people should not be allowed to work until ten o'clock at night. This would involve a serious interference with home life and the health of the worker, and would be a bad principle to perpetuate in a legislative enactment. On behalf of the Government, the Home Secretary was persuaded to say that the specified hours, 6 A.M. and to 10 P.M., are not "immutable," and it is believed the Government, if vigorously pressed, would be willing to alter the hours to 7 A.M. and 8 P.M. respectively. Towards the close of the debate the Home Secretary made a valuable concession by adding an amendment which will prevent the issue of an Order for the two-shift system if representations are made by a majority of the employers and employed in the industry concerned.

The problem of women in industry is one that affects the public health from many vital points of view. The modern outlook is involving a large absorption of women in independent labour in the factories and workshops, and in protecting them and classifying the conditions under which they are to be allowed to work, the State will be doing an essential public service. Addressing a gathering under the auspices of the National Union of Societies for Equal Citizenship, Miss Ashley recently pointed out that before the war there were rather more than two million women in industry and one million in other occupations. Of the former number, a million and a-half, she said, were in the textile and clothing trades.

In all probability that number has greatly increased since the war, and though they now work under considerably improved conditions, much remains to be done before we shall approach the practical ideals of an enlightened community.

